# Is a 3-Minute Knee MRI Protocol Sufficient for Daily Clinical Practice? A SuperResolution Reconstruction Approach Using AI and Compressed Sensing

**DOI:** 10.3390/diagnostics15101206

**Published:** 2025-05-09

**Authors:** Robert Hahnfeldt, Robert Terzis, Thomas Dratsch, Lajos Maximilian Basten, Philip Rauen, Johannes Oppermann, David Grevenstein, Jan Paul Janßen, Nour El-Hoda Abou Zeid, Kristina Sonnabend, Christoph Katemann, Stephan Skornitzke, David Maintz, Jonathan Kottlors, Grischa Bratke, Andra-Iza Iuga

**Affiliations:** 1 Department of Diagnostic and Interventional Radiology, Faculty of Medicine and University Hospital Cologne, University of Cologne, 50923 Cologne, Germany; 2 Orthopaedic Surgery and Traumatology, Faculty of Medicine and University Hospital Cologne, University of Cologne, 50923 Cologne, Germany; 3 GHO-Orthopädisch-Traumatologische Praxis Mainz, 55116 Mainz, Germany; 4 Philips GmbH Market DACH, 22335 Hamburg, Germany; 5 Röntgenpraxis Bergheim, 50126 Bergheim, Germany

**Keywords:** magnetic resonance imaging, artificial intelligence, musculoskeletal radiology, super resolution

## Abstract

**Objectives:** The purpose of this study was to assess whether a 3-min 2D knee protocol can meet the needs for clinical application if using a SuperResolution reconstruction approach. **Methods:** In this prospective study, a total of 20 volunteers underwent imaging of the knee using a 3T MRI scanner (Philips Ingenia Elition X 3.0T, Philips). The imaging protocol, consisting of a fat-saturated 2D proton density sequence in coronal, sagittal, and transverse orientations, as well as a sagittal T1-weighted sequence, was acquired with standard and ultra-low resolution. The standard sequences were reconstructed using an AI-assisted Compressed SENSE method (SmartSpeed). The ultra-low-resolution sequences have been reconstructed using a vendor-provided prototype. Four experienced readers (two radiologists and two orthopedic surgeons) evaluated the sequences for image quality, anatomical structures, and incidental pathologies. The consensus evaluation of two different experienced radiologists specialized in musculoskeletal imaging served as the gold standard. **Results:** The acquisition time for the entire protocol was 11:01 min for standard resolution and 03:36 min for ultra-low resolution. In the overall assessment, CS-SuperRes-reconstructed sequences showed slightly improved accuracy and increased specificity compared to the standard CS-AI method (0.87 vs. 0.86 and 0.9 vs. 0.87, respectively), while the standard method exhibited a higher sensitivity (0.73 vs. 0.57). Overall, 24 out of 40 pathologies were detected in the ultra-low-resolution images compared to 26 in the standard images. **Conclusions:** The CS-SuperRes method enables a 2D knee protocol to be completed in 3 min, with improved accuracy compared to the clinical standard.

## 1. Introduction

Diseases affecting the musculoskeletal system are highly prevalent and represent a significant health challenge worldwide [1,2,3,4]. A substantial part of these diseases involves the knee joint, with knee pain being a prevalent condition affecting approximately 25% [5,6,7]. Over time, the prevalence of knee pain has increased and with an aging population; an increase in knee osteoarthritis is also expected with a projected rise of approximately 40% by 2025 [1] compared to the early 2000s. Thus, joint pain ranks as the fifth most common reason for a doctor’s visit [8,9].

Magnetic resonance imaging (MRI) has emerged as a valuable tool for noninvasive imaging of the knee, thanks to its superior ability to visualize soft tissue [10]. In 2021, in the United States, 108 MRI examinations per 1000 inhabitants were recorded, with 58 taking place in hospitals and 50 within the outpatient sector. On the other hand, in Germany, Europe, up to 158 MRI examinations per 1000 inhabitants were recorded in the same year, with 23 being performed in hospitals and 135 in outpatient settings [11]. This reflects the high and continuously rising demand of MRI examinations, especially in outpatient care.

An MRI is known to have varying durations, which can range from a few minutes to over an hour, depending on the type of examination and the complexity of the protocol [12,13,14]. Pezzotti et al. introduced a novel approach to reduce the acquisition time by developing a deep-learning-based image reconstruction technique (Adaptive-CS-NET) [15] using a convolutional neural network (CNN) [15]. This CNN (SmartSpeed; Philips Healthcare, Best, The Netherlands) allows for the reconstruction of images acquired with Compressed SENSE based on variable density undersampling patterns; it is applied prior to coil combination and effectively removes noise from images [16,17]. A recently developed deep-learning-based prototype adds a series of CNNs to increase the matrix size and therewith the sharpness of the images. These types of networks are known as SuperResolution networks [18,19] and have led to significant reductions in scan duration for knee examinations. Multiple research groups have successfully implemented accelerated protocols that reduce acquisition times to sub-5-min intervals [20,21], with some protocols achieving scan times of less than 3 min while maintaining diagnostic image quality [22,23]. These developments represent a notable improvement in MRI efficiency without compromising the subjective assessment of image quality parameters that are critical for clinical diagnosis. In this study, we investigated whether the new reconstruction method (CS-SuperRes) can generate diagnostically usable sequences while reducing the acquisition time of a 2D knee MRI protocol to 3 min. Unlike most previous research, our investigation is among the few that assessed not only diagnostic image quality but also quantified radiologist confidence in their interpretations. This additional measure provides important insights into the clinical applicability of accelerated MRI protocols beyond standard quality metrics.

## 2. Material and Methods

### 2.1. Study Population

This prospective single-center study was carried out in accordance with the ethical standards in the 1964 Declaration of Helsinki and its later amendments. It was approved by the institutional review board, and it was registered in the German Clinical Trials Register (DRKS0002415). Prior to the examination, all participants provided their informed consent.

The inclusion criteria for the study specified that participants must be 18 years of age or older, have no recent history of knee-related pain within the past 6 months, and have not undergone any previous knee surgery. Exclusion criteria were applied, which included pregnancy and the presence of implanted MR-conditional devices. Volunteers were recruited exclusively through institutional advertisements. A total of 20 volunteers were ultimately scanned and included in the study from December 2022 to January 2023.

### 2.2. MRI Acquisition

For image acquisition, a dedicated knee coil (transmit/receive, 16 channels) was used in conjunction with a whole-body 3.0 T MRI system (Philips Ingenia Elition X 3.0T, Philips Healthcare, Best, The Netherlands). All participants were positioned supine on the examination table with their feet placed first. The field-of-view (FOV) encompassed the entire knee joint, including the distal femur and the proximal tibia and fibula, for all sequences. Following the recommendations of the German Society for Musculoskeletal Radiology (DGMSR) and the European Society of Musculoskeletal Radiology (ESSR), the imaging protocol comprised standard sagittal, coronal, and transverse 2D proton density (PD) sequences with fat saturation, as well as one sagittal T1-weighted sequence [22,23]. All sequences were acquired at two different resolutions: standard resolution and ultra-low resolution (for resolution details: Table 1).

### 2.3. MRI Reconstruction

The standard resolution sequences were reconstructed using the commercially available AI-algorithm (SmartSpeed, Philips Healthcare, Best, The Netherlands). These sequences are currently the clinical standard and will henceforth be described as the standard sequence. The ultra-low-resolution sequences were reconstructed using a recently developed super resolution method (CS-SuperRes). This new reconstruction algorithm consists of a series of convolutional neural networks (CNNs): Adaptive-CS-Net and Precise Image (Philips Healthcare, Best, The Netherlands), an AI-model applied to remove ringing artifacts and to replace the traditional zero-filling strategy to increase the matrix size and therewith the sharpness of the images. This network is trained on pairs of low- and high-resolution data with crops in the frequency domain to induce ringing. The implementation of data consistency checks are used to match the resulting data in the frequency domain (k-space) with the corresponding measured data.

### 2.4. Subjective Image Analysis

The subjective evaluation of the images was performed by two radiologists (PR, a board-certified radiologist with 8 years of experience, specialized in musculoskeletal imaging; and LB, a radiology resident with 5 years of experience) and two orthopedic surgeons, specialized in knee surgery (DG and JO with 8 and 12 years of experience, respectively). To ensure consistency, the four readers first conducted a consensus reading on knee MRI images that were not included in the evaluation. As part of the consensus reading, each reader conducted an independent analysis of all images.

The ground truth was established by two independent, experienced radiologists (both specialized in musculoskeletal imaging: GB, with 11 years of experience; and AII, with 8 years of experience). In the first step, each radiologist (GB and AII) independently reviewed and evaluated the images, blinded to the reconstruction type. Cases that deviated by more than 1 point were reviewed again—similar to an interdisciplinary tumor board—and a consensus was reached.

For the analysis, a self-developed software tool was used, where readers were presented with the PD sequences in standard and ultra-low resolution. All images were devoid of labels and randomly assigned to two different datasets. The readers had the possibility to adjust the window width and level settings to their preference. To avoid biases, a period of six weeks passed between the reading of each dataset for all readers.

Readers were asked to assess various anatomical structures: anterior ligament (ACL), posterior ligament (PCL), collateral ligaments (MCL and LCL), bone, menisci, and cartilage; and they were asked to identify the potential pathologies of each structure. Finally, the readers were asked to rate how confident they were in their assessment on a Likert scale from 1 to 5 (1 = unsure, 5 = sure).

### 2.5. Objective Image Analysis

Signal-to-noise ratio (SNR) and contrast-to-noise ratio (CNR) measurements were conducted by a radiology resident with one year of experience (RH) using the Picture Archiving and Communication System (PACS) installed at the institution (Impax EE R20, Agfa Healthcare, Version 12). The measurements involved manually placing regions of interest (ROI) in various anatomical structures of the knee joint: meniscus (anterior horn of the lateral meniscus), bone (distal femur), and synovial fluid. Measurements were only conducted in areas confirmed to be free of pathological conditions. ROIs measured 3 ± 0.1 mm^2^ for the meniscus, 94 ± 0.7 mm^2^ for the bone and 3 ± 0.1 mm^2^ for the synovial fluid. SNR calculations followed the methodology used in a previous study by Lee et. al., which involved dividing the average signal intensity (SI) value by the standard deviation (SD) of the tissue [24]. Additionally, CNR values for bone-fluid were calculated using an equation described in previous studies: [13,25].

$$(SI\;a-SI\;b)/\sqrt{\left({SD\;a}^{2}+{SD\;b}^{2}\right)}$$

*SD* = Standard Deviation; *SI* = Signal Intensity; *a* = Tissue a; *b* = Tissue b.

### 2.6. Statistical Analysis

All statistical analyses were conducted using GraphPad Prism version 9.0.1 for Mac OS X (GraphPad Software) as well as RStudio version 1.2.5033 for Mac OS X using the ggplot package. Contingency tables were used to assess the radiologists’ detection performance. Two-way repeated measures ANOVAs were performed to analyze the effects of the resolution (standard and ultra-low resolution) and reconstruction method (SmartSpeed vs. CS-SuperRes) on objective image quality measure including signal-to-noise ratio and contrast-to-noise ratio. Furthermore, Dunnett’s multiple comparison test was used to compare all sequences to the reference sequences (SmartSpeed). The Mann–Whitney U test was used to assess the overall confidence. All post-hoc tests were adjusted for multiple comparisons. The data are reported as mean ± standard deviation. A *p*-value below 0.05 was considered statistically significant. A priori sample size calculation was performed using G*power 3.1.9.7. based on previous findings related to acceleration techniques in knee imaging [21]. A minimum of 19 volunteers were required to detect a difference of 0.2 points on the Likert scale with a standard deviation of 0.3, alpha 0.05, and a power of 0.8 [13].

## 3. Results

### 3.1. Study Population

Twenty healthy volunteers were included (twelve men and eight women; mean age: 31.6 ± 8.9 years, range: 21–57 years; mean weight: 78.4 ± 12.4 kg, range: 57–103 kg).

### 3.2. MRI Acquisition

The standard protocol demonstrated a total acquisition time of 11 min and 1 s, while the ultra-low resolution with the CS-AI method achieved a reduced acquisition time of approximately 3 min and 36 s. The analysis revealed sequence-specific scan time reductions ranging from 55.9% to 73.4% compared to the standard protocol (Table 1).

### 3.3. Subjective Image Analysis

**Figure 1:**
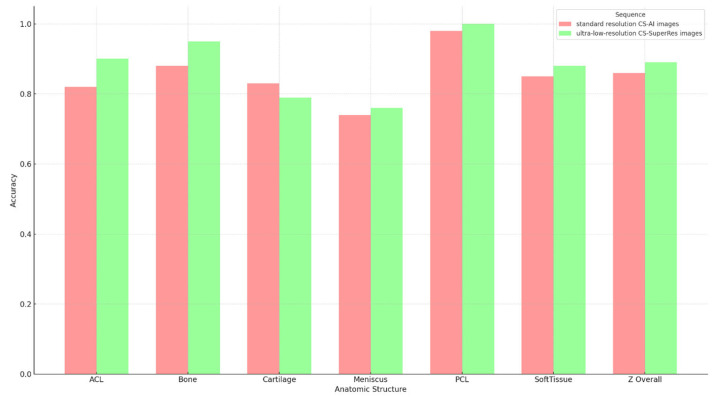
Accuracy of anatomic structures. Comparison of the accuracy between standard resolution CS-AI images (sequence A) and ultra-low-resolution CS-SuperRes images (sequence B). Overall, better accuracy was observed in the ultra-low-resolution CS-SuperRes images compared to the standard resolution. However, for the overall image quality, no significant difference was observed (*p*-value: 0.130). Legend: CS-AI = Smart Speed; CS-SuperRes = Compressed Sensing and a new deep-learning-based super resolution reconstruction approach.

In the assessment of the sequences, the accuracy, sensitivity, and specificity of potential pathologies were evaluated for all evaluated anatomical structures. Except for cartilage, all other structures showed higher accuracy with ultra-low-resolution CS-SuperRes compared to standard sequences (Figure 1). Similarly, a higher specificity for pathologies was shown for the ultra-low-resolution CS-SuperRes reconstructions compared to the standard sequences, except for the meniscus (e.g., bone: 0.984 vs. 0.906; Table 2). Overall, the ultra-low-resolution CS-SuperRes showed improved accuracy (0.875 vs. 0.846; Table 2) and increased specificity (0.9 vs. 0.875; Table 2) compared to the standard sequence. In contrast, the standard resolution showed better sensitivity for all anatomical structures, except bone (e.g., Cartilage: 0.75 vs. 0.688; Table 2). However, it must be noted that no significant difference in the accuracy was identified in any anatomical structure (*p*-value in all sections > 0.05; for the exact *p*-values, see Table 2).

**Figure 2:**
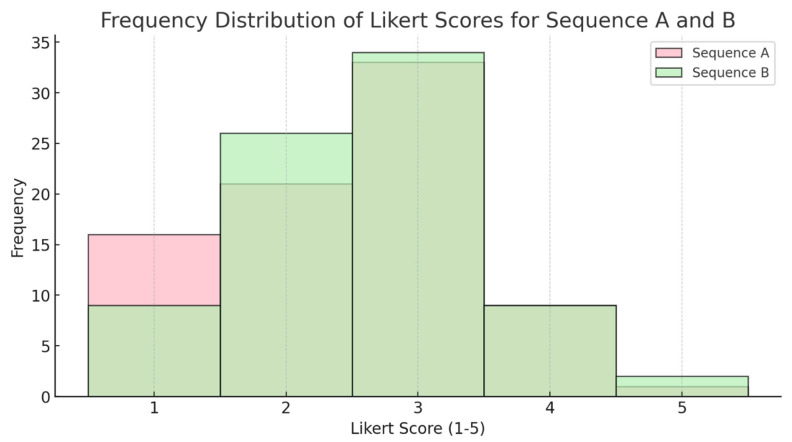
Assessment of overall confidence. Comparison of the overall confidence on a Likert Scale (1 = unsure; 5 = sure) between standard resolution CS-AI images (sequence A) and ultra-low-resolution CS-SuperRes images (sequence B). The median for sequence A is 3.0, and the median for sequence B is also 3.0. The *p*-value from the Mann–Whitney U test is 0.451, indicating that there is no significant difference between the two sequences.

**Figure 3:**
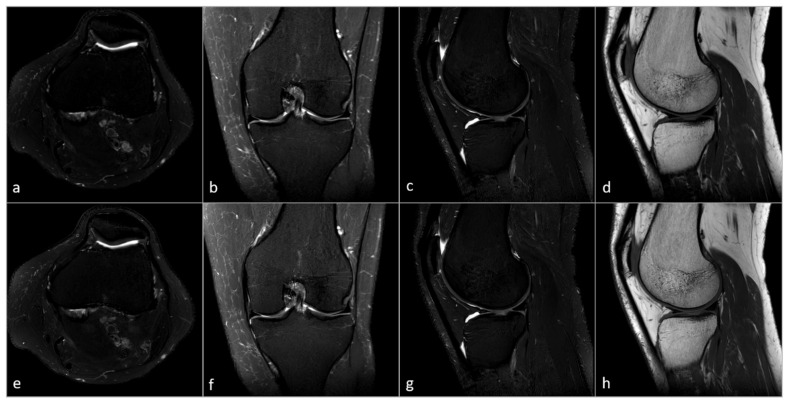
Example of a complete 2D MRI knee protocol of one volunteer. Top = Standard resolution sequences, reconstructed with CS-AI (11 min 1 s): (**a**) PD-SPAIR transversal (Scan time: 230 s); (**b**) PD-SPAIR coronal (Scan time: 178 s); (**c**) PD-SPAIR sagittal (Scan time: 160 s); (**d**) T1 sagittal (Scan time: 93 s). Bottom = Ultra-low-resolution sequences, reconstructed with CS-SuperRes (3 min 36 s): (**e**) PD-SPAIR transversal (Scan time: 79 s); (**f**) PD-SPAIR coronal (Scan time: 54 s); (**g**) PD-SPAIR sagittal (Scan time: 42 s); (**h**) T1 sagittal (Scan time: 41 s). Legend: PD = Proton Density; SPAIR = Spectral Attenuated Inversion Recovery; CS-AI = Smart Speed; CS-SuperRes = Compressed Sensing and a new deep-learning-based super resolution reconstruction approach (Precise Image); s = seconds.

In the standard sequences, 26 out of a total of 40 pathologies were detected, while with the CS-SuperRes reconstruction, 24 pathologies were detected (Table 3). In the rating of the confidence in assessing the images, no significant differences between the standard method and the CS-SuperRes method was observed (*p*-value 0.541; Figure 2). In Figure 3, you can see a complete knee protocol both in the standard method and in the accelerated method. The acquisition time of the old protocol is 11 min and 1 s, while the new protocol requires 3 min and 36 s. Another example can be seen in Supplementary Figure S1.

**Figure 4:**
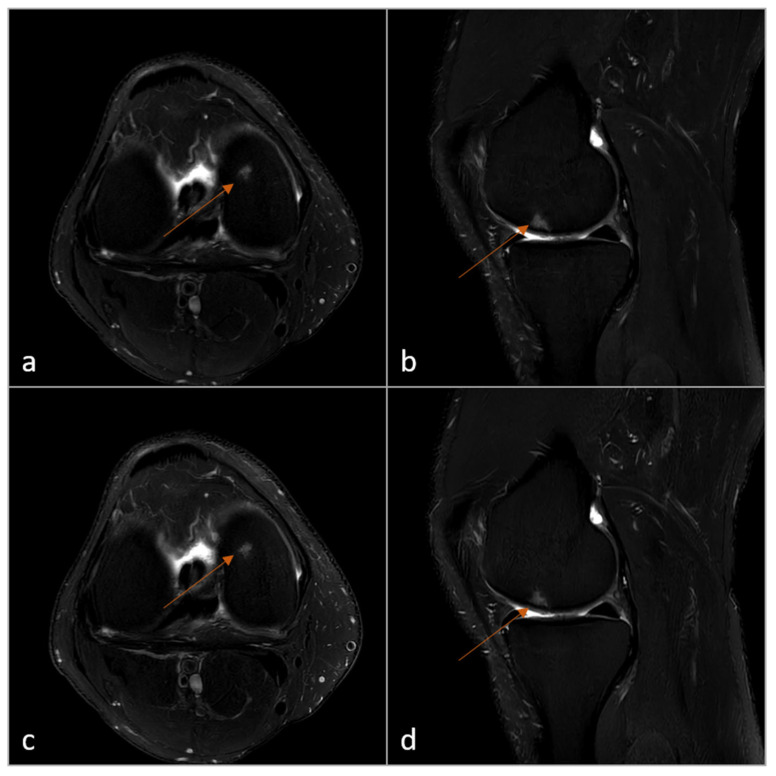
Example of incidental finding in the femur (bone edema, arrow). Top = Standard resolution sequences, reconstructed with CS-AI: PD-SPAIR transversal (**a**); PD-SPAIR sagittal (**b**). Bottom = Ultra-low resolution sequences, reconstructed with CS-SuperRes: PD-SPAIR transversal (**c**); PD-SPAIR sagittal (**d**). The acquisition time for the transversal sequence (**c**) was 79 s and for the sagittal sequence (**d**) 42 s. Legend: PD = Proton Density; SPAIR = Spectral Attenuated Inversion Recovery; CS-AI = Smart Speed; CS-SuperRes = Compressed Sensing and a new deep-learning-based super resolution reconstruction approach (Precise Image); s = seconds.

**Figure 5:**
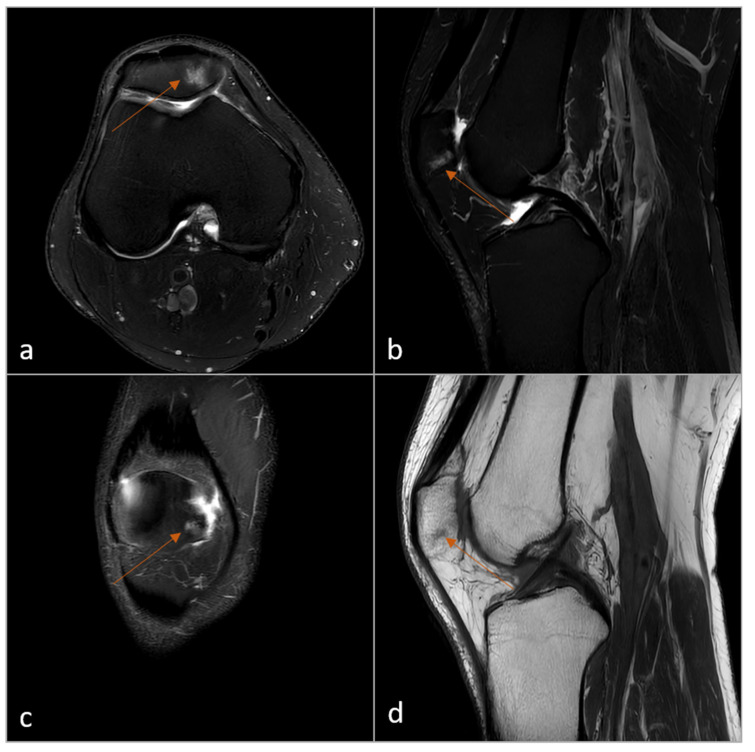
Example of a complete 3-min 2D knee protocol of one volunteer with an incidental finding in the patella (bone edema, arrow). (**a**) PD-SPAIR transversal; (**b**) PD-SPAIR sagittal; (**c**) PD-SPAIR coronal; (**d**) T1 sagittal. All sequences were acquired with the ultra-low-resolution CS-SuperRes protocol (acquisition time: 03:36 min). Legend: PD = Proton Density; SPAIR = Spectral Attenuated Inversion Recovery; CS-SuperRes = Compressed Sensing and a new deep-learning-based super resolution reconstruction approach (Precise Image).

As an example, Figure 4 and Figure 5 illustrate a small bone edema located in the distal femur and patella.

### 3.4. Objective Image Analysis

**Figure 6:**
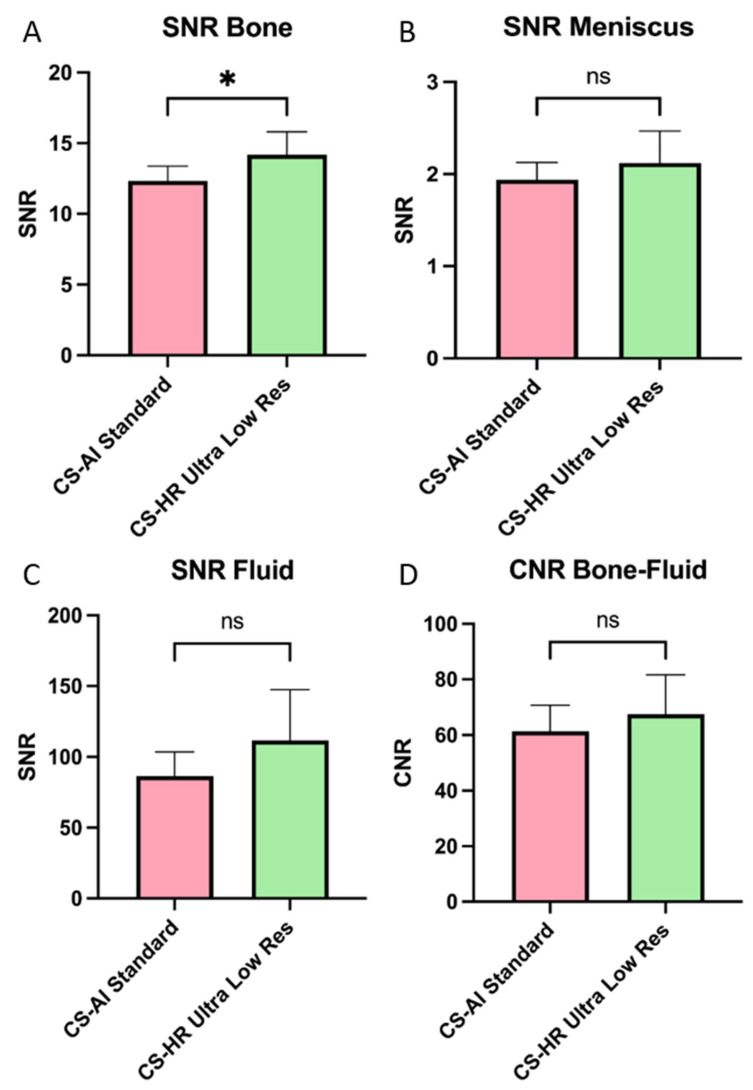
Signal-to-Noise Ratio and Contrast-to-Noise Ratio. (**A**) SNR in bone; (**B**) SNR in meniscus; (**C**) SNR in fluid; (**D**) CNR between bone and fluid. A significant difference was observed in the bone, where the SNR of the CS-SuperRes ultra-low resolution (CS-HR ultra-low resolution) is significantly higher compared to the standard method (CS-AI Standard; *p*-value < 0.05). No significant difference was observed in the SNR of the meniscus or fluid (*p*-value > 0.05). Additionally, no significant difference was observed in the CNR of bone-fluid (*p*-value > 0.05). Legend: CS-AI = Smart Speed; CS-SuperRes = Compressed Sensing and a new deep-learning-based super resolution reconstruction approach (Precise Image); * = *p*-value < 0.05; ns = no significance.

The SNR of ultra-low-resolution CS-SuperRes scans exceeded that of the standard sequences across all domains. Specifically in bone, the SNR of the ultra-low-resolution CS-SuperRes sequences significantly surpassed that of the standard sequences (Figure 5a; *p*-value: 0.022). Additionally, a higher CNR for the CS-SuperRes ultra-low-resolution sequences was observed for bone-fluid (Figure 6D; *p*-value: 0.141).

## 4. Discussion

The reduction in acquisition time not only increases the capacity for scanning more patients per unit of time but also improves the quality by minimizing the motion artifacts and enhancing patient compliance [12,14,26], making MRIs potentially also available for patients suffering from severe pain or claustrophobia.

In recent years, extensive research focused on accelerating MRI scans [24,27,28]. Iuga et al. were able to demonstrate that the acquisition time of 2D and 3D knee MRI sequences can be significantly accelerated while maintaining diagnostic quality [13]. However, higher CS factors result in violations of the Nyquist criteria, leading to artifacts such as aliasing and blurring [25]. To resolve this issue, an AI-based reconstruction method was introduced. Several studies have demonstrated further reduction in MRI sequence acquisition time using Compressed Sensing and deep learning reconstruction methods [29,30,31,32,33,34]. For instance, the study by Iuga et al. demonstrated that 2D knee MRI sequences could achieve reduced acquisition times without compromising image quality [32]. Additionally, the advantages of CS-AI extend beyond musculoskeletal imaging. Wu et al. showed that coronary MRA image acquisition times could be shortened while simultaneously enhancing image quality [30].

The purpose of this prospective study was to assess whether a 3-min 2D knee MRI protocol can meet the needs for clinical application when enhanced by a SuperResolution reconstruction approach using AI and Compressed Sensing. With the newly developed CS-SuperRes reconstruction method, the acquisition time of a 2D knee MRI protocol was reduced from 11:01 min to 03:36 min while maintain good image quality without a loss of information regarding pathologies.

Image quality was evaluated by comparing it to our institution’s standard, a commercially available, AI-aided adaptive-CS-Net reconstruction. Recent studies have demonstrated that Adaptive-CS-NET enables the more efficient and faster acquisition of MRI sequences [32,35]. In terms of musculoskeletal imaging [34,36], particularly in knee imaging, studies showed that good image quality can be obtained despite considerably faster acquisitions [32,33].

Our study shows that the combination of an ultra-low-resolution acquisition and the CS-SuperRes reconstruction allows for even faster acquisitions with high accuracy in depicting anatomical structures and increased specificity for pathologies. Overall, the shortened protocol did not significantly underperform the standard protocol, showing image accuracy ratings slightly higher compared to the standard images. Furthermore, out of 40 incidental pathologies to be detected, 26 were discovered using the standard and 24 using the CS-SuperRes sequences. The results are consistent with recently published studies. Terzis et al. demonstrated that low-resolution knee MRI sequences reconstructed with CS-SuperRes reduced acquisition time without sacrificing image quality. Bischoff et al. showed accelerated scan times of T2 sequences and improved image quality in prostate imaging using deep-learning-aided reconstruction.

The results are also supported by the objective evaluation, where the calculated SNR and CNR values of the 3-min protocol were overall higher compared to those of the standard protocol. Nevertheless, SNR and CNR values in the assessment of images reconstructed using AI should be interpreted with caution [31].

On the one hand, the addressed 3-min 2D knee MRI protocol brings the scan time of a 2D knee MRI much closer to that of a CT examination. Reducing the scan duration has several well-known benefits, including the ability to perform a greater number of examinations within a given timeframe, improving patient comfort, and minimizing the risk of motion-related image artifacts [37,38,39]. Especially for patients with claustrophobia, a shortened scan time can be advantageous, as it may improve overall tolerance for MRI examinations, enabling the access to MR imaging and therefore timely treatment for this group of patients. In a time when radiology is facing an increasing number of MRI requests, AI-based super resolution helps by significantly reducing the acquisition time of MRI sequences, thereby improving patient comfort and enhancing economic outcomes.

On the other hand, the number of performed MRI examinations per institution and day would no longer be dependent on the scan time of the examination, but rather on the institution’s logistics and time organization between individual examinations.

Some limitations of this study must be addressed. One limitation of this study is that the ground truth was established by only two readers. Nevertheless, both readers are highly experienced specialists in musculoskeletal radiology. Additionally, in standard clinical practice, radiological images are typically interpreted by a single radiologist, suggesting that our methodology represents an optimal approach within the constraints of practical clinical reality. Only a single MR scanner was used in this study, raising questions about its generalizability to other devices and manufactures. While a limited number of studies have demonstrated that certain AI-based reconstructions exhibit cross-scanner transferability [40], further investigation is warranted to establish the robustness of these techniques across varying hardware configurations, field strengths, and acquisition parameters. In the future, we intend to assess the new AI-based reconstruction method at several centers. Another limitation is the small cohort size and the initial selection of the healthy subjects. Only highly compliant volunteers were scanned. Consequently, it remains uncertain whether comparable image quality and diagnostic performance could be achieved in clinical populations with reduced compliance, such as patients experiencing pain, anxiety, or those with cognitive limitations. Nevertheless, some of the volunteers showed incidental but more subtle pathological findings, the majority of which were detected in both the standard and 3-min protocols. However, the concern that AI can produce artificial images that can mask pathologies has just partially been addressed in our study, because of the small number of pathological findings. Efforts to address this are made through the methodology used, which utilizes a multiscale network to ensure data uniformity and prevent the loss of information, but further studies addressing a greater number of MRI scans with a focus on patients are essential. What is not addressed in this study, but is very important to analyze, are the effects of shortened scan times on the daily routine of radiologists and radiologic technologists, and how reduced scan times change the organization and workflow in MRI examinations. This needs to be determined in further studies. A further limitation concerns the SNR as an objective measurement variable for image quality. It has already been shown that the SNR is tissue-dependent and may have large inter-patient variability. In future studies, one could consider using other methods such as image texturing as a feature for evaluating image quality [41].

In this study, we were not only able to demonstrate that a 2D knee protocol can be acquired in just over three minutes (as others demonstrated before), but we also showed that there is no relevant difference in the detection of incidental pathology. Certain research groups have already demonstrated that accelerated acquisition protocols can maintain diagnostic quality comparable to standard protocols, with validation through arthroscopic correlation [20,42]. Additionally, to our best knowledge, our study demonstrates for the first time that radiologists and orthopedic surgeons show no difference in their confidence levels during interpretation. In conclusion, a 3-min 2D knee MRI protocol reconstructed with CS-SuperRes does not significantly underperform the standard protocol in terms of subjective overall image quality and the accuracy of anatomical structures, potentially allowing for MRI scan times to become similar to CT times in the near future.

Key points

AI-based SuperResolution reduced 2D knee MRI acquisition time to 3 min 36 s.

AI-based SuperResolution sequences showed slightly higher accuracy and specificity than standard sequences.

AI-based SuperResolution sequences identified slightly fewer pathologies (24 vs. 26).

## Figures and Tables

**Table 1:** MR acquisition parameters for the different sequences of the evaluated standard and ultra-low-resolution protocol.

	PD SPAIR						T1 TSE	
	Coronal		Transversal		Sagittal		Sagittal	
Sequence	Standard	Ultra-Low Resolution	Standard	Ultra-Low Resolution	Standard	Ultra-Low Resolution	Standard	Ultra-Low Resolution
Echo time [ms]	45	45	50	50	50	50	12	12
Repetition time [ms]	2618	2248	3105	2840	2673	2104	724	823
Flip angle [deg.]	90	90	90	90	90	90	90	90
Field of view [mm]	160 × 160	160 × 160	150 × 150	150 × 150	160 × 160	160 × 160	160 × 160	160 × 160
Slice thickness [mm]	3	3	3	3	3	3	3	3
Number of slices	27	27	36	36	27	27	30	30
Gap [mm]	3.3	3.3	3.3	3.3	3.3	3.3	3.3	3.3
Acquisition voxel size [mm]	0.38 × 0.55	0.6 × 0.8	0.38 × 0.53	0.55 × 0.69	0.38 × 0.51	0.65 × 0.85	0.3 × 0.43	0.55 × 0.75
Reconstruction voxel size [mm]	0.22 × 0.22	0.22× 0.22	0.22 × 0.22	0.22 × 0.22	0.22 × 0.22	0.22 × 0.22	0.22 × 0.22	0.23 × 0.23
Turbo factor/Echo train length	15	16	15	15	15	15	7	7
CS-AI factor	2.5	2.5	2.5	2.5	3	3	3	3
Scan time [s]	178	54	230	79	160	42	93	41
Saved scan time [s]	0	124	0	151	0	118	0	52
Scan time reduction [%]	0	69.6	0	65.6	0	73.4	0	55.9

Legend: CS-AI = Smart Speed; PD = Proton Density; SPAIR = Spectral Attenuated Inversion Recovery; TSE = Turbo Spin-Echo.

**Table 2:** Accuracy, sensitivity, and specificity of anatomic structures and potential pathologies.

Anatomic Structure	Accuracy A	Sensitivity A	Specificity A	Accuracy B	Sensitivity B	Specificity B	*p* Value
ACL	0.825	NA	0.825	0.9	NA	0.9	0.170
Bone	0.875	0.75	0.906	0.95	0.813	0.984	0.099
Cartilage	0.825	0.75	0.844	0.788	0.688	0.813	0.610
Meniscus	0.7375	0	0.776	0.763	0	0.803	0.702
PCL	0.975	NA	0.975	0.988	NA	0.988	0.563
Soft Tissue	0.838	0.5	0.855	0.863	0	0.908	0.431
Overall	0.846	0.65	0.864	0.875	0.6	0.9	0.130

Comparison of the accuracy of anatomic structures as well as the sensitivity and specificity for potential pathologies (A = standard method; B = CS-SuperRes method). Overall improved specificity was found in the CS-SuperRes-reconstructed sequences compared to the standard method (0.864 vs. 0.9). Conversely, there was a slightly better sensitivity for potential pathologies in the standard method compared to the accelerated method (0.65 vs. 0.6). However, no significant difference was observed in any category (*p*-value: 0.130).

**Table 3:** Potential pathologies per anatomical structures.

Anatomic Structure	A (True Positive/Total Number of Pathologies	B (True Positive/Total Number of Pathologies
Bone	12/16	13/16
Cartilage	12/16	11/16
Meniscus	0/4	0/4
Soft tissue	2/4	0/4
Total	26/40	24/40

Listing of the discovered pathologies, sorted by anatomical region and sequence type (A = CS-AI sequences; B = ultra-low-resolution CS-SuperRes sequences). In total, 26 out of 40 pathologies were detected in the CS-AI sequences; 24 were found in the ultra-low-resolution CS-SuperRes sequences.

## Data Availability

The data presented in this study are available on request from the corresponding author. The data are due to privacy and ethical restrictions not publicly accessible.

## Supplementary Materials

The following supporting information can be downloaded at: https://www.mdpi.com/article/10.3390/diagnostics15101206/s1, Figure S1: Example of a complete 2D MRI knee protocol; Legend: Example of a complete 2D MRI knee protocol of one volunteer. Top = Standard resolution sequences, reconstructed with CS-AI: (a) PD-SPAIR transversal; (b) PD-SPAIR coronal; (c) PD-SPAIR; T1 sagittal Bottom= Ultra-low resolution sequences, reconstructed with CS-SuperRes: (e) PD-SPAIR transversal; (f) PD-SPAIR coronal; (g) PD-SPAIR sagittal; (h) T1 sagittal.

## Abbreviations

AI	Artificial Intelligence
CS	Compressed Sensing
MRI	Magnetic resonance imaging
CNN	Convolutional neural network
FOV	Field-of-view
SNR	Signal-to-noise ratio
CNR	Contrast-to-noise ratio
PD	Proton Density
ACL	Anterior cruciate ligament
PCL	Posterior cruciate ligament
MCL	Medial collateral ligament
LCL	Lateral collateral ligament
SI	Signal intensity
SD	Standard deviation
ROI	Region of interest
