# Supplementary material for: Is a 3-Minute Knee MRI Protocol Sufficient for Daily Clinical Practice? A SuperResolution Reconstruction Approach Using AI and Compressed Sensing
Source: Diagnostics (Basel). 2025 May 9;15(10):1206. doi: 10.3390/diagnostics15101206 (PMC12109732; doi:10.3390/diagnostics15101206)
Supplement: Supplementary file 1 [file diagnostics-15-01206-s001.zip › diagnostics-3441529-supplementary.pdf]

Figure S1. Example of a complete 2D MRI knee protocol

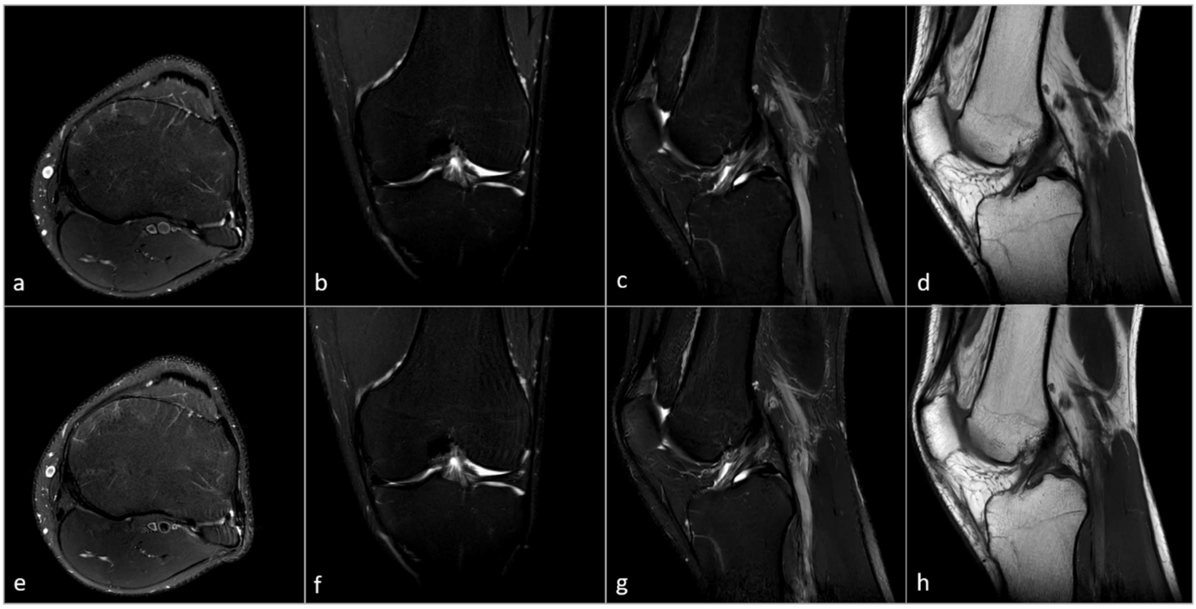

Example of a complete 2D MRI knee protocol of one volunteer. Top= Standard resolution sequences, reconstructed with CS-AI: ***:*** *(a) PD-Spair transversal; (b) PD-Spair coronal; (c) PD-Spair; T1 sagittal Bottom= Ultra-low resolution sequences, reconstructed with CS-SuperRes: (e) PD-Spair transversal; (f) PD-Spair coronal; (g) PD-Spair sagittal; (h) T1 sagittal.*
